# Ethics of a Mixed Board of Medical Examiners; Letter from Prof. N. S. Davis, M. D.

**Published:** 1896-07

**Authors:** 


THE ETHICS OF A MIXED BOARD: FATHER DAVIS' OPINION.—It will be remembered that in the May number of the *Texas Medical News*, Dr. McLaughlin, the editor, who is an ex-president of the Texas Medical Association, says: "We do not consider it any more unethical to co-operate with homeopaths on a medical board than we do on a church or school board." On this issue, the writer, the senior editor of the "Red Back," took issue with him, and held that the former association is in a *professional capacity*, and the latter a social or political function; and that as the Code is intended to regulate the conduct and intercourse of a professional man in his every professional relation—to serve on a medical board with those whose "sectarian calling makes them quacks" is a breach of the spirit, if not of the letter of the Code.

As it is a matter of grave importance to the medical profession, we appealed to Dr. N. S. Davis, the father of the American Medical Association, who is generally regarded as an exemplar of both the letter and spirit of the Code, and a wise and just expounder of its principles. Dr. Davis did us the honor to very promptly and very kindly give us his views, and it affords us much satisfaction to present his letter, herewith, as written, and with his permission:

CHICAGO, ILL., June 16, 1896.

65 RANDOLPH STREET.

*F. E. Daniel, M. D., Editor:*

DEAR SIR:—Your letter asking my opinion whether the *restrictions* on the conduct of physicians imposed by the Code of Medical Ethics, prohibited a physician from serving on a State board of health, or on a State board of medical examiners for license to practice, with homeopathic, eclectic, or other irregular practitioners, is received. I think a careful reading of the entire Code will find nothing in it directly or fairly applicable to this question. The *restrictions* in Section 1, under the head of "Duties in Regard to Consultations," certainly apply only to consultations concerning the sick and their management. The *restrictions* in Section 2, under the head of "Duties for the Support of the Professional Character," apply only to the examination and the signing of certificates or diplomas to facilitate the *graduation* of medical students intending to practice some exclusive or irregular system of medicine, either by private preceptors or professors in medical schools. State boards of health and State boards of medical examiners are created and their duties defined by State laws, and I find nothing in the Code of Medical Ethics either restricting or regulating the conduct of their members. Indeed, I think no such State boards were in existence when the Code of Ethics was framed. The proper public duties of members of such boards do not necessitate the discussion of exclusive medical theories or dogmas, and consequently no ethical barriers have been erected against the acceptance of membership by any regular physician without regard to the medical creed of other members. The Illinois State Board of Health, created in 1877, is also the State Board of Medical Examiners, and has always been composed of four regular physicians, two homeopaths and one eclectic. And similarly mixed boards exist in several other States, while others have three State boards, one for each, so-called, school of medicine. While I thus find nothing in the existing Code of Ethics prohibiting a regular physician from acting as a member of a mixed board of medical examiners, I certainly do not think it wise or proper for a physician or a medical society to ask for, or in any way advocate the enactment of any law founded on the assumption that the great body of the medical profession constitute a *school of medicine*, to be designated as *Allopathy*. First, because no such school exists, and never has existed, except in the fanciful mind of Hahneman and his followers. Consequently, its use in the enactment of laws is a misrepresentation of the profession, and strongly calculated to perpetuate the popular errors of past centuries. Second, because the only legitimate object of State boards of medical examiners is to secure for the people competent and skillful practitioners of medicine and surgery.

Consequently such boards should recognize but one standard of qualification for all applicants of whatever "school," and the appointing power should select the members solely on account of their known integrity and high professional attainments, and not because they belonged to this sect or that. For there is certainly no more propriety in making laws recognizing sectarianism in medicine than there is in religion. The title, Doctor of Medicine, is broad enough and definite enough to cover the widest scope of personal liberty in medical practice, and every intelligent practitioner should promptly resent being called or classed as an *Allopath* or any other *path*.

Yours truly,

N. S. Davis.

Notwithstanding there is nothing in the letter of the code prohibiting it,—for the reason—as suggested, that at the time there were no examining boards for license, it is a fair assumption, and a legitimate deduction that if it is contrary to the letter and spirit of the code to examine a person and sign a certificate of qualifications to enable him to *graduate* in homeopathy, it certainly does violence to the spirit of the code for a physician to "examine a person and sign a certificate of qualification" to *practice* homeopathy. That conclusion can hardly be avoided; if the act mentioned is *unethical*, to examine and license this same student to *practice*, after he has graduated, can be no less unethical! That is a proposition which requires no defense.

The position of the editor of the "Red Back" on the ethics of a mixed board is thus sustained and endorsed by the highest and most distinguished authority, as well as the position that it is unbecoming the dignity of the medical profession to ask for such legislation, involving as it does, the recognition of homeopathy and the confession that we accept the designation derisively bestowed by Hahneman—"allopathic school of medicine." We are much gratified to be permitted to lay the views of this venerable Father in Medicine before our readers for their guidance.

The bill providing for a mixed board of medical examiners should *not be presented to the legislature;* it is a *stultification*, and we trust that every physician who reads this—who has proper regard for the honor and dignity of his profession will work to accomplish its suppression or withdrawal.

